# Effect of the Plastein Reaction in Presence of Extrinsic Amino Acids on the Protective Activity of Casein Hydrolysate against Ethanol-Induced Damage in HHL-5 Cells

**DOI:** 10.3390/foods8040112

**Published:** 2019-03-29

**Authors:** Li-Ying Bo, Jia-Nan Pang, Chun-Li Song, Tie-Jing Li

**Affiliations:** 1 College of Light Industry, Liaoning University, Shenyang 110036, China; boliying1746@126.com; 2 Faculty of Food Quality and Safety, Qiqihar University, Qiqihar 161006, China; songchunlilily@sina.com; 3 Key Laboratory of Dairy Science, Ministry of Education, Northeast Agricultural University, Harbin 150030, China; jiananpang2018@163.com

**Keywords:** casein hydrolysate, plastein reaction, HHL-5 cells, antioxidant capacity, in vitro activity

## Abstract

Casein hydrolysates (CH) were prepared using papain and modified by the plastein reaction (CH-P) in the presence of extrinsic phenylalanine (CH-P-Phe) or tryptophan (CH-P-Trp). The in vitro protective activity of CH and its modified products against ethanol-induced damage in HHL-5 cells was investigated. The results showed that the modification by the plastein reaction reduced the amino group content of CH. However, the modification by the plastein reaction in the presence of extrinsic amino acids could enhance the antioxidant, proliferative, cell cycle arresting, and anti-apoptosis activity of CH. Biological activities of CH and its modified products in the HHL-5 cells varied depending on the hydrolysate concentration (1, 2, and 3 mg/mL) and treatment time (24, 48, and 72 h). Generally, higher biological activities were found after cell treatment with CH or its modified products at concentration of 2 mg/mL for 48 h compared to other treatments. In addition, CH modified in the presence of tryptophan (CH-P-Trp) showed higher biological activity than that modified in the presence of phenylalanine (CH-P-Phe). Based on the obtained results, it can be concluded that casein hydrolysates with enhanced biological activity and potential health benefits can be produced by papain and the plastein reaction with the incorporation of extrinsic amino acids.

## 1. Introduction

Millions of people in our world consume alcohol or alcoholic beverages for their physiological and psychological effects. However, moderate alcohol consumption may provide health benefits such as promoting blood circulation and reducing burden of heart disease. In addition, alcohol and alcohol beverages may provide some nutrients such as amino acids and vitamins to the human body. However, consuming too much alcohol for long time is harmful to the human health and is ranked among the top five disease risks worldwide [1,2]. The consumption of too much alcohol may cause different health problems such as cardiovascular disease, damage to the central nervous system, and bone diseases [3]. At present time, alcoholic liver disease (ALD) is one of the common chronic liver diseases and is considered a major cause of fibrosis, cirrhosis, liver cancer, and death [4]. Alcoholic liver disease may heal if patients stop drinking with the intake of nutritional supplements as well as drug treatments. However, medications for treatment of alcoholic liver disease may cause side effects. Therefore, there is a need for developing of natural ingredients or functional foods for treatment of patients with alcoholic liver disease without adverse effects.

Food protein derived hydrolysates and peptides with lower molecular weights are well-known for possessing various biological activities and effects such as antioxidant, anti-hypertensive, and mineral-binding. For example, peptides obtained from enzymatic hydrolysates of different protein sources have shown various physiological regulations such as scavenging free radicals, anti-ageing effects, and lipid peroxidation inhibition [5,6]. During the last few decades, different peptides with antioxidant properties have been derived from different plant and animal proteins such as casein [7], corn protein [8], fish protein, and other proteins [9,10]. Antioxidants are considered important nutraceuticals which possess many other health benefits [11,12,13]. The antioxidant activity of food protein-derived peptides can be enhanced by some treatments such as the plastein reaction and the protease hydrolysis reaction. Recently, many studies have revealed that the plastein reaction-modified CH can increase the in vitro antioxidant activities of hydrolysates [14,15,16]. In addition, extrinsic amino acids could be added into the reaction system to supplement the products enhanced properties such as antihypertensive and cytoprotective effects [17,18]. However, the influence of the plastein reaction on the potential protective effect of protein hydrolysates against ethanol-induced hepatocytes is not well investigated.

The objective of this work was to produce functional hydrolysate by hydrolysis of casein with papain and the modification by a papain-hydrolyzed plastein reaction in the presence of extrinsic amino acids, including phenylalanine (Phe) and tryptophan (Trp). Biological activities including the effects on cell proliferation, cell cycle arrest, and apoptosis prevention were investigated in hepatocytes.

## 2. Materials and Methods

### 2.1. Materials and Reagents

Casein was purchased from Beijing Aoboxing Biotechnologies Inc. (Beijing, China). Papain and 1,1-Diphenyl-2-picryl-hydrazyl (DPPH) were purchased from Sigma Chemical Co. (St. Louis, MO, USA). The HHL-5 hepatocytes were purchased from Cell Bank of the Chinese Academy of Sciences (Shanghai, China). The fetal bovine serum (FBS) was purchased from Wisent Inc. (Montreal, QC, Canada). DMEM: F12 (1:1) medium were purchased from Hyclone Chemical Co. Ltd. (St. Louis, MO, USA). Cell Counting Kit-8 (CCK-8) was purchased from Dojindo Molecular Technologies, Inc. (Kyushu, Japan). Cell Cycle Analysis Kit and Annexin V-FITC Apoptosis Detection Kit were purchased from Beyotime Institute of Biotechnology (Shanghai, China). Propidium iodide (PI), trypsin-EDTA, and Rnase A reagent were purchased from Solarbio Science and Technology Co. Ltd. (Beijing, China). Ultrapure water used in this study was generated using a Milli-Q Plus water purification system (Millipore Corporation, New York, NY, USA). Other chemicals used were of analytical grade.

### 2.2. Preparation and Modification of Casein Hydrolysate

Casein hydrolysates (CH) were prepared as described by Tomita et al. [19] with slight modifications. Briefly, a casein protein sample of 5.0 g was dissolved in 100 mL water, and the pH of the casein solution was adjusted to 6.5 using 1 mol/L NaOH solution, and then papain was added into the casein solution (2.2 KU/g protein). The hydrolysis reaction was performed at 45 °C for 4 h. After that, the mixture was adjusted to pH 4.6 with 1 mol/L HCl, heated at 80 °C for 15 min to terminate the hydrolysis reaction, cooled to 20 °C, and then centrifuged at 5000 rpm for 20 min. The radical scavenging activity on DPPH (2,2-diphenyl-1-picrylhydrazyl) of the collected supernatant was determined and then stored at −20 °C for further use.

The preparation of modified CH products in the presence of extrinsic Phe and Trp as well as the plastein reaction conditions of CH in the presence of extrinsic Phe (i.e., CH-P-Phe) and Trp (i.e., CH-P-Trp) were carried out as described in our previous study [17]. Briefly, the plastein reaction was performed by the addition of papain to CH solution under stirring. The reaction conditions were as follows: Enzyme/substrate ratio of 3.0 KU/g protein, CH concentration of 50 g/L, temperature of 30 °C, pH 5.0, and incubation time of 6 h. CH-P-Phe and CH-P-Trp were prepared for the case of extrinsic Phe and Trp added at 0.74 mol/mol free amino groups of CH. CH-P was prepared without extrinsic Phe and Trp addition, while CH-P+Phe and CH-P+Trp were prepared by mixing CH-P and extrinsic Phe and Trp with inactivated papain. All products were freeze-dried with a freeze-dryer (ALPHA 1–4 LSCplus, Marin Christ, Osterode, Germany), and stored at −20 °C until analyses.

### 2.3. Assays of Protein and Free Amino Contents

Nitrogen content of Casein, CH, CH-P, and modified products were determined according to the Kjildahl method [20] and converted to estimated protein content using a nitrogen-to protein conversion factor of 6.25.

Based on the result of peptide condensation, free amino groups (-NH_2_) of reaction substrates were decreased during the plastein reaction [21]. The free amino group (-NH_2_) contents of casein and its modified products were determined using the o-phthaladehyde (OPA) assay [22]. The OPA solution was prepared by dissolving 2.0 g of sodium dodecyl sulfate in 30 mL of 0.4 mol/L sodium borate buffer (pH 9.5). Then, 200 μL β-mercaptoethanol, 80 mg OPA in 1 mL ethanol (ETOH), and borate buffer were mixed to obtain a final solution volume of 100 mL. A volume of 3 mL OPA reagent was mixed with 3 mL sample solution and kept for 5 min before measuring the absorbance at 340 nm with an UV-2401PC spectrophotometer (Shimadzu, Kyoto, Japan). Leu solutions (0–36 μg/mL) were used to obtain a standard curve, and the results were expressed as -NH_2_ mmol/g protein.

### 2.4. Antioxidant Activity Assays

#### 2.4.1. Ferrous Reducing Power Activity Assay

The ferrous reducing power of samples was analyzed by Canabady-Rochelle et al. [23]. Hydrolysate solution was diluted to 0.2 mg/mL with 2.0 mL phosphate buffer (0.2 mol/L, pH 6.6), and 2.0 mL of 1% (w/v) potassium ferricyanide was added. The solution was mixed and incubated at 50 °C for 30 min and then cooled to room temperature. The mixture solution was then centrifuged for 10 min at 3000 rpm and 2.0 mL of 10% (v/v) trichloroacetic acid was added. Ferric chloride (0.5 mL, 0.1%) and distilled water (2 mL) was mixed with the supernatant (2 mL). Then the absorbance of the solution was determined at 700 nm by a UV spectrophotometer (UV-2401PC, Shimadzu, Tokyo, Japan). The data in duplicate were analyzed using Graphpad Prism software (PRISM 5.0; GraphPAD Software Inc., San Diego, CA, USA).

#### 2.4.2. DPPH Radical-Scavenging Activity Assay

The DPPH radical-scavenging activity of CH and its modifiers (CH-P, CH-P+Phe, CH-P-Phe, CH-P+Trp, and CH-P-Trp) was assayed by Santos, Brizola, and Granato [24] with minor modifications, while scavenging activity on hydroxyl radicals was calculated as described by Chung, Osawa, and Kawakishi [25]. All analyses were performed at a UV-2401PC spectrophotometer (Shimadzu, Kyoto, Japan).

### 2.5. Cell Culture Conditions and Ethanol-Induced HHL-5 Damage Assay

The HHL-5 hepatocytes were cultured in a 1:1 DMEM/F-12 culture medium containing 10% FBS, 100 units/mL penicillin, and 100 μg/mL streptomycin, and maintained at 37 °C in a humidified atmosphere containing 5% CO_2_.

The cells were seeded in 96-well plates at a density of 4 × 10^3^ cells per well in a 200 μL medium. After incubation of 24 h, the medium was removed, and a fresh medium containing ETOH at concentrations of 0, 100, 200, 300, and 400 mmol/L was added with treatment times of 24, 48, and 72 h. The cells treated without the medium containing ETOH were used as a blank control. Then, 100 μL CCK-8 solution (10 μL CCK-8 in 90 μL medium) was added into each well, followed by incubation for 3 h. Optical density values were measured at 450 nm using a microplate reader (Bio-Rad Laboratories, Hercules, CA, USA).

### 2.6. Cell Viability Assay

The cells were seeded in 96-well plates at a density of 1 × 10^4^ cells per well in a 200 μL medium and incubated at 37 °C for 24 h in a medium containing 10% FBS. The medium was replaced with 200 μL of a fresh medium containing CH and its modifiers at concentrations of 1, 2 and 3 mg/mL for 24, 48 h, and 72 h and then each well added in 300 mmol/L ETOH for 48 h. The cells treated with a normal medium were a negative control group, and the cells treated with the medium containing 0.5 mg/mL BHA were a positive control group. After incubation of all the cell groups, a 100 μL CCK-8 solution (10 μL CCK-8 in 90 μL medium) was added into each well, followed by incubation for 3 h. Optical density values were measured at 450 nm using a microplate reader (Bio-Rad Laboratories, Hercules, CA, USA), and used to calculate viability value.

### 2.7. Cell Cycle Assay

The cell cycle distribution was assayed by the classical propidium iodide (PI) staining method and flow cytometry analysis according to kit manufacturer’s instructions. Briefly, the cells were seeded in 100-mm cell culture dish at 2 × 10^5^ cells for 24 h in a 10 mL medium containing 5% FBS. Then, the cells were exposed to CH and its modifiers at 2.0 mg/mL for 48 h. The cells without hydrolysate treatment were used as a negative control. The cells were collected by trypsin-EDTA, washed twice with phosphate-buffered saline (PBS, 10 mmol/L, pH 7.3), suspended in 70% ETOH at −20 °C overnight, washed with the cold PBS again, and then re-suspended in a solution containing 10 μL Rnase A (20 μg/mL) and 25 μL PI (50 μg/mL) for 30 min at 37 °C in the dark. The cells in a medium containing 5% FBS were used as a negative control. Proportion of the cells in G0/G1-, S-, and G2/M-phases were analyzed with the ModFit software (Verity Software House, Topsham, ME, USA), and measured using a flow cytometry (FACS Calibur, Becton Dickson, San Jose, CA, USA).

### 2.8. Cell Apoptosis Assay

Fluorescent dyes, Annexin V-FITC, and PI were used to detect the apoptotic and necrotic cells. Annexin V-FITC identifies early and late apoptotic cells, while late apoptosis and necrotic cells are stained by PI. The protocol used in the present analysis was recommended by the kit manufacturer. The cells at a density of 2×10^4^ cell per well were cultured in 6-well plates with a 2 mL medium for 24 h and then treated with the CH and its modifiers at 2 mg/mL for 48 h, followed by the addition of the induction agent ETOH (300 mmol/L) and incubation for 48 h. The cells without a hydrolysate treatment were used as a negative control, while the cells treated with BHA were used as a positive control. After that, the cells were harvested by trypsin-EDTA, washed with PBS twice, re-suspended in a 400 μL Annexin V-FITC binding buffer containing 5 μL PI and 10 μL Annexin V-FITC for 30 min at 20 °C in the dark, and analyzed by the flow cytometry (FACS Calibur, Becton Dickson) to measure the intact (Q3), early apoptotic (Q4), late apoptotic (Q2), and necrotic (Q1) cell proportions.

### 2.9. Statistical Analysis

Data obtained from triplicate were statistically analyzed and expressed means ± standard deviations (SD). One-way analysis of variance (ANOVA) with Duncan’s multiple range tests was performed by SPSS 16.0 software (SPSS Inc., Chicago, IL, USA). Differences were considered significant at level of 95% (*p* < 0.05).

## 3. Results

### 3.1. The Free Amino Group Content

The reduction in the content of -NH_2_ is an important feature of the plastein reaction. The -NH_2_ contents of casein hydrolysate (CH) and its modified products (CH-P, CH-P+Phe, CH-P-Phe, CH-P+Trp, and CH-P-Trp) were determined, and the results are shown in Table 1. The -NH_2_ content of the modified product CH-P decreased by 0.207 mmol/g compared with that of CH, indicating the occurrence of the plastein reaction, i.e., peptide condensation. The -NH_2_ contents of the CH-P+Phe and CH-P+Trp products were found to be 1.739 and 1.742 mmol/g protein, respectively. The -NH_2_ contents of CH-P-Phe and CH-P-Trp reduced by 0.388 and 0.394 mmol/g protein compared with those of CH-P+Phe and CH-P+Trp, respectively. This indicates that extrinsic Phe and Trp were successfully incorporated into the modified products, CH-P-Phe and CH-P-Trp.

### 3.2. Antioxidant Activity

The antioxidant activity of CH and its modified products were measured by different in vitro assays, and the results are presented in Table 2. The DPPH radical-scavenging activity, reducing power, and hydroxyl radicals scavenging activity of CH increased after the plastein reaction. On the other hand, the modification of CH by the plastein reaction with the addition of amino acids resulted in products with higher antioxidant activities compared with those of products modified by the plastein reaction only. However, DPPH scavenging activity, reducing power, and hydroxyl radicals scavenging activity were found to be higher for CH-P+Trp and CH-P-Trp than those of CH-P+Phe and CH-P-Phe products. The obtained results indicate that casein hydrolysate with antioxidant activity can be successfully produced by papain, and its antioxidant activity can be enhanced by the plastein reaction and incorporation of amino acids.

### 3.3. Proliferative Effect

**Figure 1:**
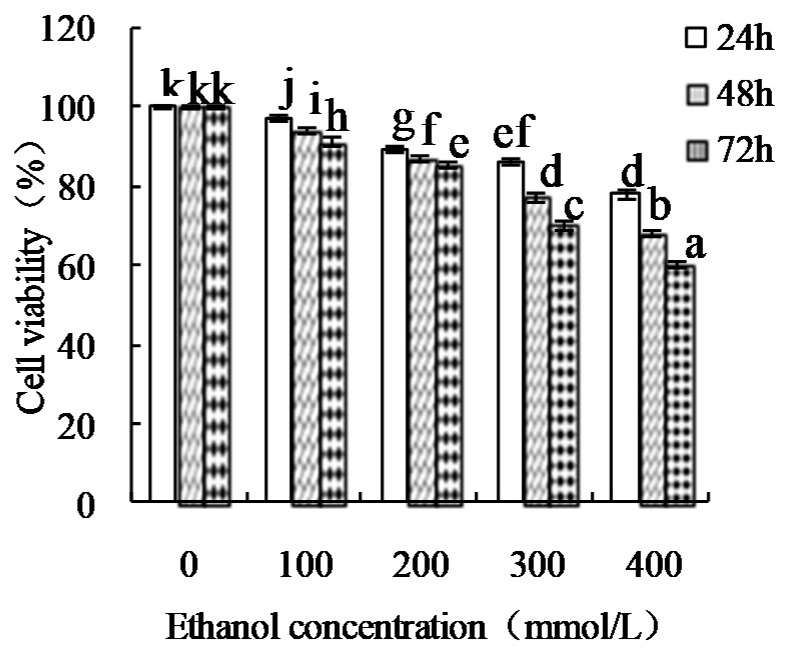
Viability of the HHL-5 cells at different ethanol (ETOH) concentrations and treatment times. Different letters above the columns indicate that the means of different groups were significantly different (*p* < 0.05) by one-way analysis of variance.

The damage effect of ETOH on the HHL-5 cells were measured, and the results were shown in Figure 1. ETOH showed a significant inhibitory effect in the HHL-5 cells depending on concentration and treatment time. Treatment with an ETOH concentration of 300 mmol/L for 48 h was chosen for preparing ETOH-induced damage HHL-5 hepatocytes.

The proliferative effect of CH and its modified products were determined in ETOH-induced HHL-5 hepatocytes, and the results are presented as cell viability percentage in Table 3. It should be noted that the cell proliferation was very obvious after treatment of ETOH-induced HHL-5 hepatocytes by butyl hydroxyl anisd (BHA) with a viability percentage ranged from 127.5 to 136.2% after incubation for 48 h. From data presented in Table 3, it can be seen that the cell viability percentage of CH is lower than that of its modified products, indicating that the plastein reaction and the incorporation of amino acids enhanced the proliferative effect of CH. On the other hand, the cell viability percentage varied depending on hydrolysate concentration (1, 2, or 3 mg/mL) and incubation time (24, 48, or 72 h). Higher cell viability percentages were found after cell treatment with CH-P-Phe or CH-P-Trp at a concentration of 2 mg/mL for 48 h compared with other treatments.

### 3.4. The Effect on the Cell Cycle

**Figure 2:**
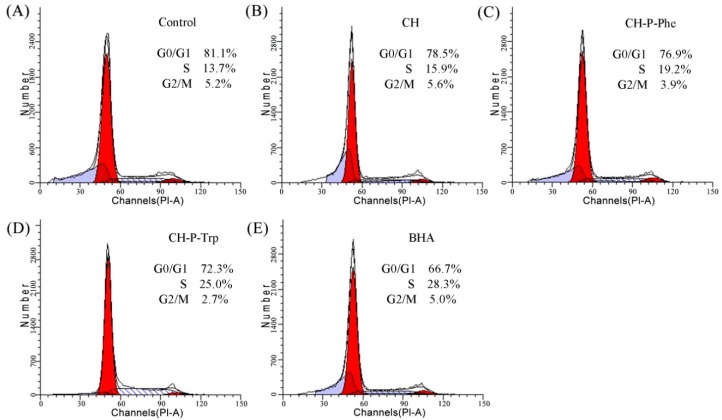
Cell cycle distribution in the HHL-5 hepatocytes treated without (**A**) or with CH (**B**), CH-P-Phe (**C**), CH-P-Trp (**D**), and BHA (**E**) for 48 h, respectively.

To investigate the in vitro protective activity of CH and its modified products in the cells via accelerating cell cycle progression, the flow cytometry technique was used to measure cell proportions of the G0/G1-, S- and G2/M-phases, using cells treated with a medium containing BHA as a positive control and cells treated with a normal medium as a negative control (Figure 2). Compared to the negative control group, CH, CH-P-Phe, and CH-P-Trp showed a slight effect on the cell proportion of the G2/M-phase but enhanced the cell proportion of the S-phase and reduced the cell proportion of G0/G1-phase cells. On the other hand, CH-P-Trp revealed a more potent cells arresting ability in the S-phase (25.0%); which is higher than those of CH-P-Phe (19.2%) and CH (15.9%), indicating that CH-P-Trp could accelerate cell cycle progression in the S-phase. These results are consistent with the proliferative activity of CH and its modified products (Table 3).

### 3.5. Anti-Apoptosis Activity

**Figure 3:**
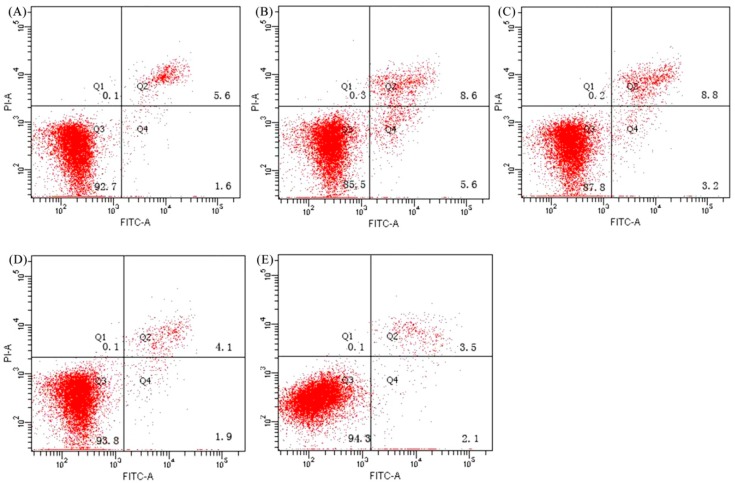
Apoptotic prevention of CH, CH-P-Phe, CH-P-Trp, and BHA in the HHL-5 hepatocytes. The cells were treated without (**A**) or with CH (**B**), CH-P-Phe (**C**)**,** CH-P-Trp (**D**), and BHA (**E**) for 48 h, respectively. Values are means of three independent assays.

The potential anti-apoptosis or apoptotic prevention effect of CH and its modified products (CH-P-Phe and CH-P-Trp) in the HHL-5 hepatocytes was determined by flow cytometry analysis (Figure 3). Cells treated with a medium containing 5% FBS were used as a negative control, while cells treated with BHA were used as a positive control. The proportion of total apoptotic cells (Q2+Q4) was measured from three independent experiments for all samples after treatment for 48 h and used to express the apoptotic prevention effect. A total apoptotic proportion of 7.2% was found for the cells of the negative control group. However, the cells in the positive control group showed lowest total apoptotic proportion of 5.6%, indicating a high apoptotic prevention effect of BHA on ETOH-damage-induced HHL-5 hepatocytes. On the other hand, total apoptotic proportions of 14.2, 12.0, and 6.0% were found after treatment of cells with CH, CH-P-Phe, or CH-P-Trp at a concentration of 2 mg/mL for 48 h, respectively. These results indicate that the CH-P-Trp at 2 mg/mL has the highest apoptotis prevention compared with CH and CH-P-Phe (*p* < 0.05). In addition, these results indicate that the plastein reaction with the incorporation of amino acids resulted in modified casein hydrolysates with higher anti-apoptosis activity. The anti-apoptosis activity of CH and its modified products is consistent with their antioxidant, proliferative, and cell cycle arresting activities.

## 4. Discussion

Many studies have found that the modification by the plastein reaction enhances biological activities of protein hydrolysates such as antioxidant activity [15], antihypertensive activity [18], and the inhibition of angiotensin converting enzyme [26]. Products modified by the Maillard reaction in the presence of extrinsic amino acids also can lead to enhancement of these biological activities [27,28]. Therefore, the application of the plastein reaction in the field of bioactive peptides is a promising technique [16]. The plastein reaction is a protease-catalysed process, and its mechanisms include condensation, transpeptidation, and physical interactions of peptides aggregates. Modified products with new amino sequences can be produced via these reaction mechanisms. It is well known that amino acid sequences and compositions play an important role in peptide bioactivity [29]. A last study had verified that pepsin-hydrolyzed by the plastein reaction with tryptophan increased in vitro activity of lactoferrin hydrolysates in human gastric cancer BGC-823 cells [30]. In the present study, the CH and CH-P at the same dose levels showed lower growth promoting activity in the HHL-5 hepatocytes; however, the modified product CH-P-Phe and CH-P-Trp showed greater activities than those of CH-P+Phe and CH-P+Trp mixtures. These results indicate that Phe and Trp incorporation as well as the plastein reaction resulted in a modified hydrolysate (CH-P-Phe and CH-P-Trp) with enhanced biological activity.

Different food components have shown in vitro protective effects in normal cells. For example, glycoprotein derived from *Laminaria japonical* showed the ability to stimulate the proliferation of IEC-6 cells on a dose-dependent manner [31]. In another study, polysaccharides from *Ganoderma lucidum* have shown obvious protective effects on gastrointestinal mucosal function, as they can promote the proliferation and migration of IEC-6 cells [32]. Moreover, hydrolysates or peptiedes derived from food proteins have possessed protective effects on different cells. Hydrolysates generated from casein showed a protective effect on hepatic HepG2 and PC12 H_2_O_2_-induced cells and promoted the growth of both cell lines [33,34]. Furthermore, bovine collagen peptides increased the proliferation of MC3T3-E1 cells [35]. Results of these studies are in a good agreement with results of this study. Results obtained from this study indicated the modification by the plastein recation and incorporation of amino acids enhanced the HHL-5 hepatocytes proliferation activity of the casein hydrolysate.

Generally, cell continuous division can promote cell rapid proliferation, and the cell cycle is an essential process of cell division. Therefore, accelerating cell cycle progression is an effective way to promote cell proliferation [36]. Many studies have shown that bioactive peptides can increase the cell cycle in certain phases. For example, peptides derived from bovine collagen protein hydrolysates could alter cell cycle distribution and increase the cell proportions of the G_2_/S phase [35]. In addition, peptides derived from soy and casein proteins by papain could enhance the cell percentage of the S-phase in osteoblastic cells [37]. In this study, CH and its modified products increased the cell cycle in the S-phase (Figure 2), which is consistent with previous studies. In addition, protein hydrolysates or bioactive peptides were found to promote cell proliferation via exerting anti-apoptosis activities in osteoblastic cells [37,38]. However, studies on apoptosis prevention activities of CH modified by the papain-catalyzed plastein reaction in the presence of extrinsic Phe and Trp in the HHL-5 hepatocytes are limited. The findings obtained in this study about anti-apoptosis activities of the CH and its modified products in the HHL-5 hepatocytes might be a novel perspective of food research.

## 5. Conclusions

The papain-generated casein hydrolysate was modified by the plastein reaction with the incorporation of phenylalanine or tryptophan. The casein hydrolysate showed higher antioxidant activity based on in vitro assays and the antioxidant activity increased after modification of the casein hydrolystae by the plastein reaction with the addition of amino acids. In addition, the casein hydrolysate and its modified products showed proliferation activity in the HHL-5 hepatocytes depending on hydrolysate concentration, amino acid type, and antioxidant activity. In addition, the casein hydrolysate and its modified products could arrest cell cycle progression in the S-phase and antagonize ETOH-induced apoptosis, shown as apoptotic prevention or reversal for the HHL-5 hepatocytes. Generally, the casein hydrolysate modified products, including CH-P-Phe and CH-P-Trp, showed higher biological activities compared to the corresponding hydrolysate. The obtained results revealed that hydrolysates or peptides can with different biological activities and potential health benefits can be produced from casein by enzymatic hydrolysis and the plastein reaction modification.

## Figures and Tables

**Table 1:** The free amino group content of different samples.

Sample	Free Amino Group Content (mmol/g)
Casein	0.523 ± 0.002 ^a^
CH	0.941 ± 0.001 ^c^
CH-P	0.734 ± 0.001 ^b^
CH-P+Phe	1.739 ± 0.002 ^f^
CH-P-Phe	1.351 ± 0.003 ^e^
CH-P+Trp	1.742 ± 0.002 ^g^
CH-P-Trp	1.348 ± 0.003 ^d^

Values are expressed as means ± SD (*n* = 3). Different superscript letters within same column indicate that means are significantly different (*p* < 0.05).

**Table 2:** Antioxidant properties of CH and its modified products.

Samples	Amino Acid Addition Level (mol/mol)	DPPH Scavenging Activity (%)	Reducing Power	Hydroxyl Radicals Scavenging Activity (%)
CH	0	35.87 ± 0.075 ^a^	0.572 ± 0.0093 ^a^	21.46 ± 0.79 ^a^
CH-P	0	36.42 ± 0.071 ^b^	0.594 ± 0.0084 ^b^	27.62 ± 0.83 ^b^
CH-P+Phe	0.74	39.56 ± 0.085 ^d^	0.673 ± 0.0087 ^d^	32.43 ± 0.84 ^d^
CH-P-Phe	0.74	38.53 ± 0.082 ^c^	0.616 ± 0.0092 ^c^	30.55 ± 0.91 ^c^
CH-P+Trp	0.74	48.63 ± 0.059 ^f^	0.724 ± 0.0095 ^f^	36.41 ± 0.92 ^f^
CH-P-Trp	0.74	46.85 ± 0.065 ^e^	0.705 ± 0.0082 ^e^	34.62 ± 0.95 ^e^

Values are expressed as means ± SD (*n* = 3). Different letters within the same column indicate that the mean values are significantly different (*p* < 0.05).

**Table 3:** Cell viability (%) of the HHL-5 hepatocytes treated with different samples for 24–72 h.

Samples	Sample Doses and Treating Times of the Cells								
	1.0 mg/mL			2.0 mg/mL			3.0 mg/mL		
	24 h	48 h	72 h	24 h	48 h	72 h	24 h	48 h	72 h
CH	104.2 ± 3.8 ^a^	110.4 ± 2.5 ^c^	105.6 ± 3.1 ^a^	106.5 ± 1.7 ^ab^	113.0 ± 2.3 ^b^	111.5 ± 2.1 ^bc^	105.1 ± 3.1 ^a^	109.6 ± 5.7 ^b^	110.8 ± 2.3 ^b^
CH-P	105.3 ± 2.1 ^a^	109.4 ± 3.3 ^bc^	107.5 ± 3.4 ^ab^	110.6 ± 4.4 ^b^	115.1 ± 3.1 ^b^	113.4 ± 3.0 ^cd^	108.7 ± 2.6 ^ab^	115.2 ± 2.4 ^bc^	113.2 ± 1.9 ^bc^
CH-P+Phe	103.4 ± 2.3 ^a^	104.2 ± 1.8 ^a^	106.4 ± 2.7 ^ab^	104.6 ± 3.2 ^a^	105.8 ± 2.4 ^a^	106.7 ± 2.5 ^a^	105.3 ± 3.2 ^a^	106.2 ± 3.4 ^a^	104.7 ± 2.4 ^a^
CH-P-Phe	107.6 ± 2.3 ^ab^	112.5 ± 4.2 ^c^	109.2 ± 2.8 ^bc^	117.6 ± 3.8 ^c^	120.2 ± 2.2 ^c^	117.3 ± 2.4 ^c^	110.6 ± 3.5 ^b^	116.7 ± 3.5 ^bc^	114.6 ± 3.3 ^bc^
CH-P+Trp	104.6 ± 3.1 ^a^	105.9 ± 3.4 ^ab^	106.8 ± 3.2 ^ab^	105.9 ± 2.6 ^a^	106.8 ± 3.6 ^a^	107.8 ± 3.4 ^ab^	105.4 ± 3.2 ^a^	106.9 ± 2.9 ^a^	105.2 ± 2.6 ^a^
CH-P-Trp	110.5 ± 4.1 ^b^	117.4 ± 3.1 ^d^	112.4 ± 3.3 ^c^	123.4 ± 3.2 ^d^	129.1 ± 4.1 ^c^	123.1 ± 2.6 ^e^	114.5 ± 3.2 ^c^	118.2 ± 3.4 ^c^	115.0 ± 2.3 ^c^

Means within the same column with different superscript letters are different significantly (*p* < 0.05).

## Abbreviations

CH	Casein hydrolysate
CH-P	Plastein reaction-modified CH in the absence of extrinsic Phe or Trp
CH-P-Phe	Plastein reaction-modified CH in the presence of extrinsic Phe
CH-P+Phe	Mixture of CH and extrinsic Phe
CH-P-Trp	Plastein reaction-modified CH in the presence of extrinsic Trp
CH-P+Trp	Mixture of CH and extrinsic Trp
CCK-8	Cell counting kit-8
EDTA	Ethylenediamine tetra-acetic acid
FBS	Fetal bovine serum
PBS	Phosphate-buffered saline
Trp	Tryptophan
Phe	Phenylalanine
Ethanol	ETOH

## References

[1] World Health Organization (2011). The global status report on alcohol and health 2011. http://www.who.int/substance_abuse/publications/global_alcohol_report/en/.

[2] Lim S.S., Vos T., Flaxman A.D., Danaei G., Shibuya K., Adair-Rohani H. (2012). A comparative risk assessment of burden of disease and injury attributable to 67 risk factors and risk factor clusters in 21 regions, 1990–2010: A systematic analysis for the Global Burden of Disease Study 2010. Lancet.

[3] Hennig M., Yip-Schneider M.T., Klein P., Matos J.M., Doyle C., Choi J., Wu H., O’Mara A., Menze A., Noble S. (2009). Ethanol-TGF-a-MEK signal promotes growth of human hepatocellular carcinoma. Food Res. Int..

[4] Minana J.B., Gomez-Cambronero L., Lloret A., Pallardo F.V., Del Olmo J., Escudero A., Rodrigo J.M., Pellin A., Vina J.R., Sastre J. (2002). Mitochondrial oxidative stress and CD95 ligand: A dual mechanism for hepatocyte apoptosis in chronic alcoholism. Hepatology.

[5] Chalamaiah M., Dinesh Kumar B., Hemalatha R., Jyothirmayi T. (2012). Fish protein hydrolysates: Proximate composition, amino acid composition, antioxidant activities and applications: A review. Food Chem..

[6] García M.C., Puchalska P., Esteve C., Marine M.L. (2013). Vegetable foods: A cheap source of proteins and peptides with antihypertensive, antioxidant, and other less occurrence bioactivities. Talanta.

[7] Díaz M., Decker E.A. (2004). Antioxidant mechanisms of caseinophosphorpeptides and casein hydrolysates and their application in ground beef. J. Agr. Food Chem..

[8] Zhou C., Hu J.L., Ma H.L., Yagoub A.E.A., Yu X.J., Owusu J., Ma H.Y., Qin X.P. (2015). Antioxidant peptides from corn gluten meal: Orthogonal design evaluation. Food Chem..

[9] Torres-Fuentes C., Contreras M.D.M., Recio I., Alaiz M., Vioque J. (2015). Identification and characterization of antioxidant peptides from chickpea protein hydrolysates. Food Chem..

[10] Hernández-Jabalera A., Cortés-Giraldo I., Dávila-Ortíz G., Vioque J., Alaiz M., Girón-Calle J., Megías C., Jiménez-Martínez C. (2015). Influence of peptides–phenolics interaction on the antioxidant profile of protein hydrolysates from Brassica napus. Food Chem..

[11] Droge W. (2002). Free radicals in the physiological control of cell function. Physiol. Rev..

[12] Lee J., Koo N., Min D.B. (2004). Reactive oxygen species, aging, and antioxidative nutraceuticals. Compr. Rev. Food Sci. Food Saf..

[13] Valko M., Leibfritz D., Moncol J., Cronin M.T., Mazur M., Telser J. (2007). Free radicals and antioxidants in normal physiological functions and human disease. Int. J. Biochem. Cell B..

[14] Zhao X.H., Li Y.Y. (2009). An approach to improve ACE-inhibitory activity of casein hydrolysates with plastein reaction catalyzed by Alcalase. Eur. Food Res. Technol..

[15] Sun H., Zhao X.H. (2012). Angiotensin I converting enzyme inhibition and enzymatic resistance in vitro of casein hydrolysate treated by plastein reaction and fractionated with ethanol/water or methanol/water. Int. Dairy J..

[16] Udenigwe C.C., Rajendran S.R.C.K. (2016). Old products, new applications? Considering the multiple bioactivities of plastein in peptide-based functional food design. Curr. Opin. Food Sci..

[17] Zhao X.H., Fu Y., Yue N. (2014). In vitro cytoprotection of modified casein hydrolysates by plastein reaction on rat hepatocyte cells. J. Food.

[18] Xu J.L., Pang J.N., Chen F.F., Li T.J., Zhao X.H. (2017). Antihypertensive activities of the plasteins derived from casein hydrolysates in spontaneously hypertensive rats. CyTA-J. Food.

[19] Tomita M., Bellamy W., Takase M., Yamauchi K., Wakabayashi H., Kawase K. (1991). Potent antibacterial peptides generated by pepsin digestion of bovine lactoferrin. J. Dairy Sci..

[20] AOAC (2005). Official Methods of Analysis of Association of Official Analytical Chemists International.

[21] Yue N., Li T.J., Zhao X.H. (2013). The impact of extrinsic amino acids and solvent fractionation on the in vitro antioxidant activity of plastein reaction-stressed casein hydrolysates. Food Technol. Biotech..

[22] Church F.C., Swaisgood H.E., Porter D.H., Catignani G.L. (1983). Spectrophotometric assay using o-phthadialdehyde on determination of proteolysis in milk and isolated milk proteins. J. Dairy Sci..

[23] Canabady-Rochelle L.L., Harscoat-Schiavo C., Kessler V., Aymes A., Fournier F., Girardet J.M. (2015). Determination of reducing power and metal chelating ability of antioxidant peptides: Revisited methods. Food Chem..

[24] Santos J., Brizola V., Granato D. (2017). High-throughput assay comparison and standardization for metal chelating capacity screening: A proposal and application. Food Chem..

[25] Chung S.K., Osawa T., Kawakishi S. (1997). Hydroxyl radical scavenging effects of species and scavengers from brown mustard (Brassica nigra). Biosci. Biotech. Bioch..

[26] Li Y.Y., Li T.J., Zhao X.H. (2010). Preparation of Alcalase-catalyzed casein plasteins in the presence of proline addition and the ACE-inhibitory activity of the plasteins in vitro. Eur. Food Res. Technol..

[27] Zhao X.H., Wu D., Li T.J. (2010). Preparation and radical scavenging activity of papain-catalyzed casein plasteins. Dairy Sci. Tech..

[28] Sun H., Li T.J., Zhao X.H. (2014). ACE inhibition and enzymatic resistance in vitro of a casein hydrolysate subjected to plastein reaction in the presence of extrinsic proline and ethanol- or methanol-water fractionation. Int. J. Food Prop..

[29] Udenigwe C.C., Aluko R.E. (2012). Food protein-derived bioactive peptides: Production, processing, and potential health benefits. J. Food Sci..

[30] Ma C.M., Zhao X.H. (2019). Pepsin-catalyzed plastein reaction with tryptophan increases the in vitro activity of lactoferrin hydrolysates with BGC-823 cells. Food Biosci..

[31] Lee M.K., Kim I.H., Chori Y.H., Nam T.J. (2015). A peptide from Porphyra yezoensis stimulates the proliferation of IEC-6 cells by activating the insulin-like growth factor I receptor signaling pathway. Int. J. Mol. Med..

[32] Chen I.H., Lin Z.B., Li W.D. (2011). Ganoderma lucidum poly-saccharidesexate-induced small intestinal damage in mice via induction of epithelial cell proliferation and migration. Acta Pharmacol. Sin..

[33] Xie N., Wang C., Ao J., Li B. (2013). Non-gastrointestinal-hydrolysis enhances bioavailability and antioxidant efficacy of casein as compared with its in vitro gastrointestinal digest. Food Res. Int..

[34] Zhang Q.X., Ling Y.F., Sun Z., Zhang L., Yu H.X., Mburu Kamau S., Lu R.R. (2012). Protective effect of whey protein hydrolysates against hydrogen peroxide-induced oxidative stress on PC12 cells. Biotechnol. Lett..

[35] Liu J.L., Zhang B., Song S.J., Ma M., Si S.Y., Wang Y.H., Xu B.X., Feng K., Wu J.G., Guo Y.C. (2014). Bovine collagen peptides compounds promote the proliferation and differentiation of MC3T3-E1 pre-osteoblasts. PLoS ONE.

[36] Blais A., Fan C., Voisin T., Aattouri N., Dubarry M., Blachier F., Tome D. (2014). Effects of lactoferrin on intestinal epithelial cell growth and differentiation: An in vivo and in vitro study. Biometals.

[37] Pan X.W., Zhao X.H. (2015). In vitro proliferation and anti-apoptosis of the papain-generated casein and soy protein hydrolysates towards osteoblastic cells (hFOB1.19). Int. J. Mol. Sci..

[38] Fu Y., Zhao X.H. (2013). In vitro responses of hFOB1.19 cells towards chum salmon (Oncorhynchus keta) skin gelatin hydrolysates in cell proliferation, cycle progression and apoptosis. J. Funct. Food.

